# Novel Grafted Hydrogel for Iron and Ammonia Removal from Groundwater: A Synthesis and Computational Chemistry Study

**DOI:** 10.3390/gels9100781

**Published:** 2023-09-25

**Authors:** H. M. Abd El-Salam, Ali M. El Shafey, Abdelouahid Samadi, Mahmoud K. Abdel-Latif

**Affiliations:** 1 Polymer Research Laboratory, Department of Chemistry, Faculty of Science, Beni-Suef University, Beni-Suef City 62514, Egypt; hanafya@yahoo.com (H.M.A.E.-S.); mly390861@gmail.com (A.M.E.S.); 2 Chemistry Department, Collage of Science, United Arab Emirates University, Al-Ain 15551, United Arab Emirates; samadi@uaeu.ac.ae; 3 Chemistry Department, Faculty of Science, Beni-Suef University, Beni-Suef City 62514, Egypt

**Keywords:** grafted hydrogel, groundwater, ammonia and iron removal efficiency, DFT, MEP, binding energy

## Abstract

Current research is moving towards iron and ammonia elimination from groundwater. Here, we are using a poly acrylic–poly acrylamide hydrogel that is grafted with 3-chloroaniline. This copolymer was synthesized by addition polymerization technique. The effects of agitation time, dosage and adsorbent temperature on the removal process sensitivity were investigated. The copolymer was described experientially and theoretically. Isothermal kinetic adsorption models are discussed. This hydrogel could be regenerated efficiently (98.3% removal of iron and 100% removal of ammonia). The density functional theory (DFT) method, using B3LYP/6-311G(d,p), and the LANL2DZ level of the theory were managed to investigate the stationary states of the grafted copolymer and the complexation energy of the hydrogel with the studied cations. DFT has been used to investigate the Natural Bond Orbital (NBO) properties to locate the most negative centers on the hydrogel. The calculated complexation energy showed hydrogel selectivity with regard to the studied cations.

## 1. Introduction

Groundwater is one of the world’s primary sources of drinking water. Iron and ammonia are present in groundwater in a dissolvable reduced state due to natural, chemical environment and human activities [1,2,3]. Metal ions in water resources cause a variety of aesthetic and operational issues, including a repulsive unpleasant taste, laundry stains and network accumulation [4,5,6,7], which are caused by the ammonia in groundwater. The chemical oxidizing of ammonia and iron is useful, but harmful byproducts and secondary pollutants limit their use [8,9,10,11]. Iron is the fourth most prevalent element, the second most abundant metal in the Earth’s crust [12] and a common part of groundwater. There are two types of iron sources in groundwater: geogeic and anthropogenic. A geogeic source is where groundwater flows from the aquifer’s soil, sand, gravel and rocks [13]. Anthropogenic sources, e.g., industrial effluents, landfill leakages, acid mine drainage and others, lead to high concentrations in the groundwater [14]. Water percolating through soil and rock dissolves iron-containing minerals and keeps them in solutions [15] that are widespread in groundwater and surface waters with a significant groundwater input [16]. Staining, disagreeable tastes and appearances come from these solutions [17]. Higher Fe concentrations hamper Fe^+2^ oxidation and cause undesired properties. In addition, the presence of iron bacteria in the water supply system alters the water smell and promotes bacteria growth in pipes. Excessive iron content in groundwater creates technical challenges, such as the failure of water supply systems, water quality degradation and the formation of unwanted incrustations in higher oxygen water, resulting in a reduction in the pipe flow cross-section [18,19,20,21]. There are no health-related recommendations for the content of iron in drinking water. Based on taste and annoyance concerns, the World Health Organization recommends that the iron concentration in drinking water be less than 0.3 mg/L [22]. Polluted water causes 80% of diseases in affluent countries, with a death toll of 10 million every year [23]. Elemental Fe is rarely found in nature because Fe^+2^ and Fe^+3^ interact with oxygen and sulfur-containing molecules, creating oxides, hydroxides, carbonates and sulfide. Oxides are the most common form in nature [24]. Ammonia removal from water is accomplished using a variety of processes [25,26,27,28]. Many techniques are employed to recover Fe compounds from groundwater [29]. Extracting iron compounds from groundwater for drinking purposes using aerobic oxidation is not sufficient due to the full oxidation of Fe^+2^ and the growth of iron bacteria in sand filters or valves, causing discoloration and turbidity. There are many hydrogels used in the removal of iron and ammonia, such as a recyclable phytate–polyaniline hydrogel [30], the sodium alginate-g-poly(sodium acrylate) hydrogel [31], the chitosan-g-poly (acrylic acid)/rectorite hydrogel composite [32] and the acrylic acid polymer hydrogel nano Fe_3_O_4_ [33]. This study used a poly (acrylate/acrylamide) grafted with 3-chloroaniline to extract iron and ammonia through the adsorption technique [34,35,36,37]. The synthesized grafted hydrogel was studied using TEM, SEM, TGA, XRD and FTIR computationally. The efficient removal of iron and ammonia from the groundwater was studied using contact time, adsorbent dosage and temperature. The sorption data were evaluated by Langmuir, Frendlich and Temkin’s models.

## 2. Results and Discussion

### 2.1. Characterization of Obtained Polymeric Samples

**Figure 1:**
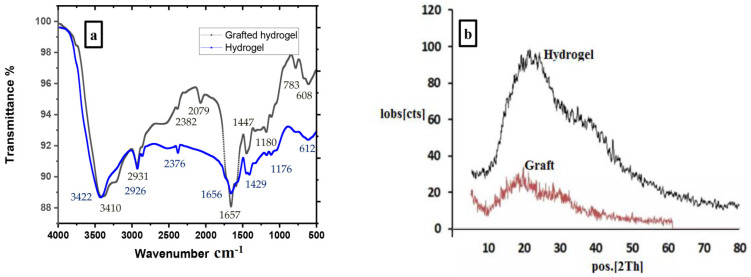
IR and XRD of both hydrogel and grafted hydrogel (**a**,**b**), respectively.

Infrared spectroscopy of the hydrogel and the graft is presented in Figure 1a. The main IR bands of the acrylamide, acrylate and 3-chloroaniline are clearly shown [38]. The C–O and C–N stretching vibrations are found between 1100 and 1200 cm^−1^ [39,40]. The CH_2_ bending appeared between 1300 and 1459 cm^−1^ [41]. The stretching vibration of C=C of benzene appeared in case of the graft at 1590 cm^−1^. The carbonyl group (C=O) stretching vibration for both hydrogel and grafted hydrogel compounds appeared at ~1656 cm^−1^ [42]. The aliphatic CH vibrations are shown after 2900 cm^−1^ [43]. The broad stretching vibrations of the OH group and the free or bonded NH_2_ group appear after 3400 cm^−1^ [44]. The stretching vibration of substituted benzene was found for the graft between 608 and 783 cm^−1^ [38], confirming the grafting process. For more details for all absorption bands and their assignments, see Table S1 (Supplementary Material).

#### 2.1.1. XRD and TGA Analysis

**Figure 2:**
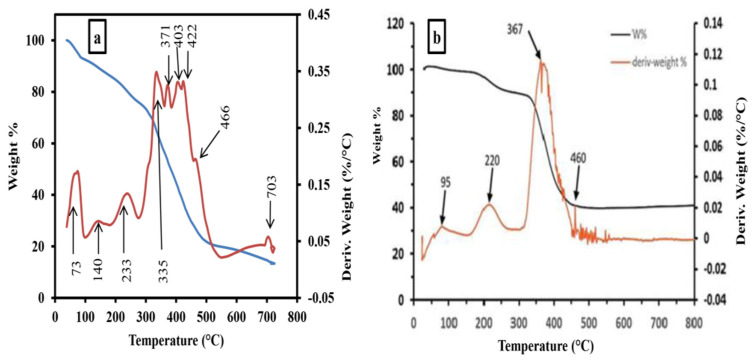
TGA of hydrogel and grafted hydrogel (**a**,**b**), respectively.

Figure 1b shows XRD of both the hydrogel and graft, indicating that the hydrogel and graft are semi-crystalline materials. Thermogravimetric analysis (TGA) for both the hydrogel and grafted hydrogel is presented in Figure 2a,b [8,45]. The temperature midpoints of the degradation are summarized in Table S2 (Supplementary Material). It is concluded that the bonded water molecules which are absorbed by the hydrogel and graft are degraded at the end of 233 °C. Also, it is clear that the quantity of absorbed water of the hydrogel is higher. Both the hydrogel and graft are thermally degradable with different stages. The progressive degradation of both hydrogel and the grafted hydrogel at ~480 °C is evident in Figure 2a,b. In addition, a higher residual quantity of the graft at ~40% is shown in Figure 2b, indicates that the grafted hydrogel is more thermally stable than the hydrogel. The TGA for both fabricated copolymers is tabulated in Table S2 (Supplementary Material). The TGA curves showed a three-stage weight loss. The hydrogel showed an initial decomposition temperature (IDT) of 230 °C and a final decomposition temperature (FDT) of 466 °C. The IDT and FDT of the grafted hydrogel were found at 220 °C and 460 °C. The first-stage weight loss of the hydrogel took place in the temperature range of 70 to 230 °C with about 21% weight loss, and in the range of 90 to 220 °C with about 13% weight loss in case of the grafted hydrogel. This may be attributed to the removal of moisture and bonded water loss. The second-stage decomposition of hydrogel started at 230 °C and ended at 466 °C with about 72% weight loss, and between 220 and 460 °C with about 60% for the grafted hydrogel. This weight loss may be due to the degradation of the hydrogel and the graft. The third-stage weight loss was shown above 460 °C which showed a complete degradation, and the remaining residue was 10% of hydrogel and 40% of grafted hydrogel. Thus, the grafted hydrogel showed a higher thermal stability than the hydrogel.

#### 2.1.2. SEM and TEM Analysis

**Figure 3:**
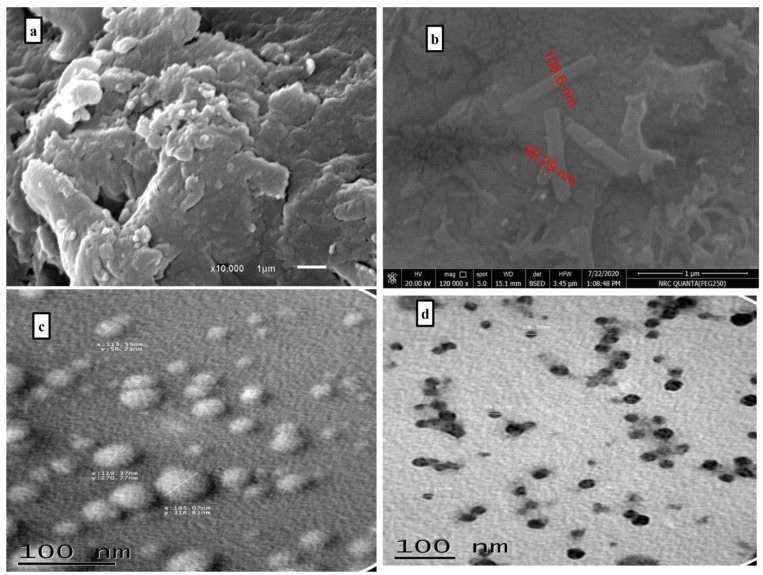
SEM images: (**a**) PAC-PAM hydrogel; (**b**) grafted PAC-PAM hydrogel. TEM: (**c**) PAC-PAM hydrogel; (**d**) grafted PAC-PAM hydrogel.

Scanning and transition electron microscope (SEM and TEM) analyses are presented in Figure 3. SEM pictures showed that the presence of 3-chloroaniline in the structure of hydrogel leads to the filling the pores of the hydrogel and minimizes the heterogeneous surface. In addition, the particles are compacted and smooth. SEM pictures of the hydrogel and graft indicated the difference between them, where the hydrogel particles have the same shape while the graft seems to have different shapes which range from spherical to tubes. In addition, TEM pictures revealed that the grafted hydrogel particle sizes are smaller than the hydrogel. The size of hydrogel particles ranged from 113 to 195 nm; while for the graft, the particle sizes ranged from 15.28 to 24.6 nm. This difference in surface for both compounds confirmed the successful grafting process.

### 2.2. Optimized Geometries

**Figure 4:**
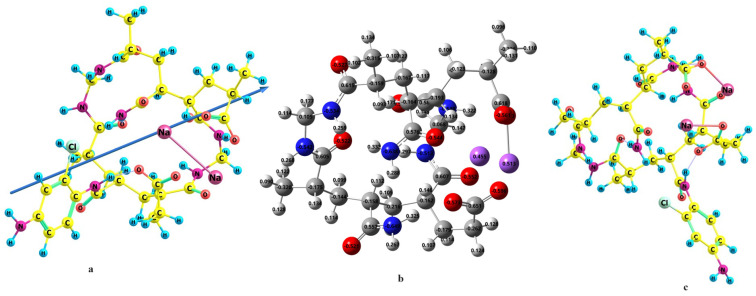
The optimized structure, of the hydrogel showing the dipole moment vector (**a**), the calculated charge density using NBO (**b**) and the optimized structure of the grafted hydrogel (**c**) at the B3LYP/6-311G(d,p) level of theory.

The optimized geometry for hydrogel with the natural charge density and graft is shown in Figure 4; hydrogel (a), charge density (b) and grafted hydrogel (c), using the B3LYP/6-311G(d,p) level of theory.

In fact, many arrangements of the monomers together were computed using the DFT method. The most stable (least steric hindrance arrangement) structure [27] was used for all calculations. Figure 4 shows the optimized structure of the copolymer with the dipole moment direction, the charge density (b) and the optimized structure of the graft (c) calculated at the B3LYP/6-311G(d,p) level of theory. As mentioned in our previous article [27], the grafting process happened in a radical cation mechanism; thus, the most positive (least negative) nitrogen atom in the copolymer attacked the para position of the chloroaniline. As shown in Figure 4, the nitrogen atom of the amino group has a −0.513 charge density; thus, it is the best place for attacking the para position of chloroaniline. The optimized structure showed the two strings of the copolymer connected by the linker. The grafting occurred on the nitrogen of the amino group on one of the strings. The aim of that article is the use of our hydrogel in water treatment, in other words, the extraction of the metal cations by the grafted copolymer. Thus, in the first place, we should find the most preferable position on the hydrogel for complexing with the positive cations.

### 2.3. Removal of Cations

#### 2.3.1. Removal of Ammonia

**Figure 5:**
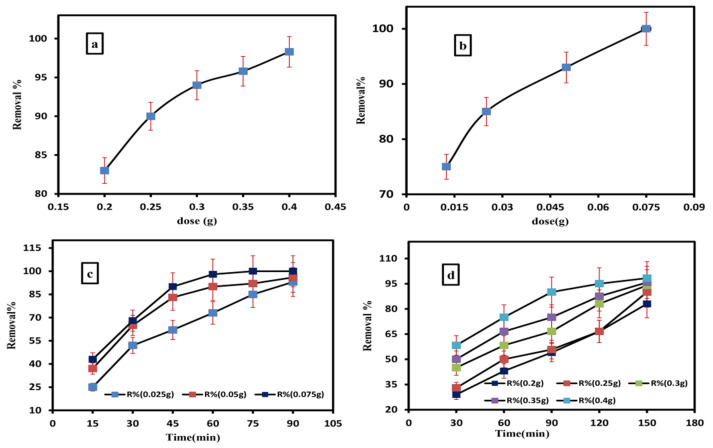
Effect of grafted hydrogel doses on iron (**a**) and ammonia (**b**) removal; the effect of contact time on the removal of ammonia (**c**) and iron (**d**).

The effect of the grafted hydrogel dose on enhancing the removal percentage of ammonia is presented in Figure 5b,c. This effect was investigated using different quantities (0.025, 0.05 and 0.075 g) of the adsorbent in 100 mL of raw groundwater for 50 min. The residual ammonia concentrations were determined using YSI 9300 and 9500 photometers at room temperature. Figure 5b,c show that 0.075 g of grafted hydrogel is enough for the complete removal of ammonia at 75 min.

#### 2.3.2. Removal of Iron

By increasing the dose of the grafted hydrogel, the percentage of iron elimination increased. this has been studied for 150 minutes using various adsorbent masses (0.2, 0.25, 0.3, 0.35, and 0.4 g) in 100 mL of raw (untreated) groundwater as shown in Figure 5a,d. The residual iron concentrations were determined using YSI 9300 and 9500 photometers at room temperature. Figure 5a,d shows that 0.4 g of hydrogel was enough for the removal of 98.3% iron at a contact time of 150 min.

The effects of contact time on the adsorption capacity of the studied materials for studied cations (ammonia and iron) were investigated. In this study we have used 0.025, 0.05 and 0.075 g for the removal of ammonia and 0.2, 0.25, 0.3, 0.35 and 0.4 g for the removal of iron by the grafted hydrogel. The adsorbents were suspended in 0.8 mg/L of ammonia and 1.2 mg/L of iron polluted solutions for different time intervals separately. After each time interval, the residual ammonia and iron concentrations were determined using YSI 9300 and 9500 photometers. All of the experiments were performed at room temperature. Figure 5c,d show that 0.075 g grafted hydrogel was enough for the complete removal of ammonia at 75 min. It was shown that 0.05 g achieved 96% at 90 min and 0.025 g achieved 93% at 90 min. Figure 5 shows that the maximum removal was achieved for all investigated quantities at a contact time of 150 min. The most effective amount of the grafted hydrogel for a high iron removal of ~98.3% was 0.4 g; however, upon reducing the adsorbent quantities, the removal percentage of iron decreased, and this can be attributed to the lowering of the graft hydrogel surface area. The removal of iron using 0.35 g is 95.8%, 0.3 g is 94%, 0.25 g is 90% and 0.2 g is 83%.

#### 2.3.3. Static Studies

Standard deviation (SD) was calculated for the removal of both iron and ammonia at optimum conditions using the following equation,

SD = √(∑(Xi − µ)^2^/N) (1)

where Xi is the removal percentage of samples, N is number of samples and µ represents the mean of the removal. The number of groundwater samples used to check the efficiency of the graft on the removal of both iron and ammonia is 5 samples (S1, S2, S3, S4 and S5). The presented sample in the figure is S1. The statics of both iron and ammonia for 5 samples are compiled in Table 1.

### 2.4. Effect of Temperature and Thermodynamics

The temperature effects on the removal of cations using grafted hydrogel were studied by adding 0.025, 0.05 and 0.075 g for ammonia removal and 0.2, 0.25, 0.3, 0.35 and 0.4 g for iron removal to raw groundwater at a range of temperatures (10, 20, 30, 40 and 50 °C) for 50 and 150 min for ammonia and iron, respectively. The residual ammonia and iron concentrations were determined using YSI 9300 and 9500 photometers. The removal (%) is plotted against temperature (Figure S1, Supplementary Material). The obtained results revealed that the effect of temperature on the removal efficiency is considered poor. Moreover, higher temperatures have a detrimental effect on the removal process, and this could be attributed to the broken physical adsorbed cations on the polymeric surface. In addition, the removal efficiency increased again at 323 K, and this was not due to adsorption but may be referred to the escaping of ammonia from the medium. While in case of iron, the increasing iron removal at 323 K may be attributed to the incorporation of iron in a phenyl ring moiety of the graft by a sandwich formation structure via π-Skelton. The thermodynamic parameters are deduced from the relations [46]:

(2) $$\Delta G^{0}=-R\mathrm{Tln}K_{c}\;$$

(3) $$\Delta G^{0}=\Delta H^{0}-T\Delta S^{0}\;\;\;\;\;$$

(4) $$\ln K_{C}=\frac{\Delta S^{0}}{R}-\frac{\Delta H^{0}}{RT}\;\;\;\;$$

where *R* is the gas constant, *T* is the absolute temperature, *K_C_*, is the Langmuir constant, Δ*H°* is the standard enthalpy and Δ*S°* is the entropy of adsorption. Δ*H°* and Δ*S°* could be estimated from the straight-line relationship between ln *K_C_* vs. *1/T* [47] (Figure S1, Supplementary Material). The calculated data are summarized in Table S3 (Supplementary Material). The thermodynamic parameters revealed that the adsorption of cations on the surface of the grafted hydrogel is exothermic and spontaneous.

### 2.5. Adsorption Isotherms

#### 2.5.1. Langmuir Isotherm

**Figure 6:**
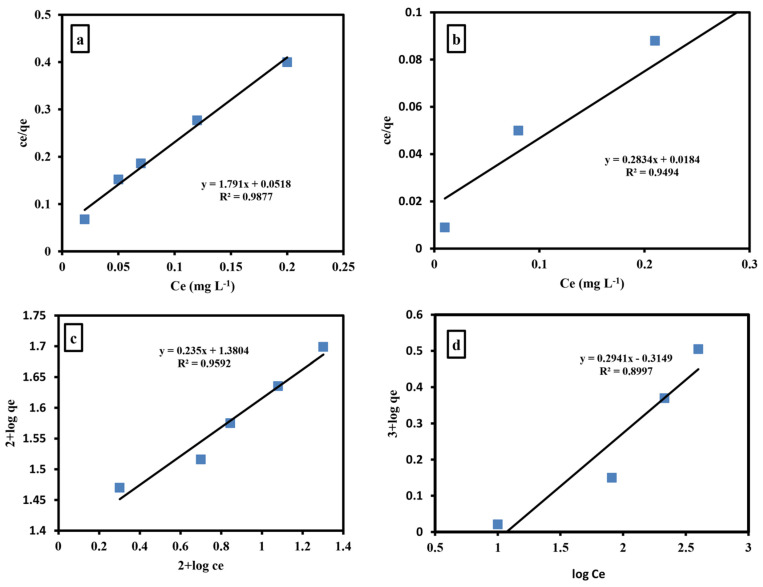
Langmuir isotherm for iron (**a**) and ammonia (**b**) removal by grafted hydrogel. Freundlich isotherm for iron (**c**) and ammonia (**d**) removal by grafted hydrogel.

The Langmuir adsorption form [48] is checked as a model according to the equation,

(5) $$\frac{C_{e}}{\;q_{e}}=\frac{C_{e}}{Q_{m}}\;+\frac{1}{Q_{m}\;b\;\;}$$

for the adsorption of cations on polymeric surfaces. The results are represented in Figure 6.

#### 2.5.2. Freundlich Isotherm

The Freundlich isotherm [49] is used in the study of the adsorption of various substances. It is utilized to examine the cations’ adsorption on the grafted hydrogel. The equilibrium results are fitted with a logarithmic form of the Freundlich model:

(6) $$\ln q_{e}=\ln K_{f}+\frac{1}{n}\ln C_{e}$$

where *K_f_* represents the adsorption capacity, n represents the heterogeneity factor and *C_e_* (mg/L) represents the adsorbate concentration.

#### 2.5.3. Temkin Isotherms

The adsorption of the studied cations on grafted hydrogel was also investigated by the Temkin isotherm. According to this isotherm, the energy of adsorption reduced linearly with surface coverage due to adsorbent/adsorbate interactions. The Temkin isotherm equation [50] is

(7) $$q_{e}=\mathrm{BTlnKT}+\mathrm{BTln}C_{e}$$

where *q_e_* is the total amount of cations adsorbed by the polymeric sample at equilibrium (mg/g) and *C_e_* (mg/L) is the adsorbate concentration at equilibrium. Freundlich constants depend on the capacity and strength of adsorption, respectively. *Q_m_* is the monolayer adsorption capacity (mg/g). B is a constant linked to the adsorption heat and it is given by B = RT/b, where b is the Temkin constant (J/mol), T is the absolute temperature (K) and R is the gas constant (8.314 Jmol^−1^K^−1^). Linear relationships are obtained upon plotting *Ce/q_e_* vs. *Ce*, ln*q_e_* vs. ln*Ce* and *q_e_* vs. ln*Ce*, and these are presented in Figure S2 (Supplementary Material). Table 2 summarizes all data. The data confirmed that the removal of cations was governed by a Langmuir model.

#### 2.5.4. Adsorption Kinetics

The adsorption mechanism of cations on the grafted hydrogel from groundwater was studied using two kinetic models, the pseudo-first-order kinetic model [51]:

(8) $$\ln\left(q_{e}-q_{t}\right)={\mathrm{lnq}}_{e}-k_{1}t$$

and the Lagergren pseudo-second order model [52]:

(9) $$\frac{t}{q_{t}}=\frac{1}{k_{2}q_{0}{}^{2}}+{\frac{t}{q}}_{e}\;\;$$

where q_t_ represents the quantity of adsorbed ions at time t (mg/g), k_1_ and k_2_ represent the first- and second-order adsorption rate constants (g/mg min), respectively. The parameters of first- and second-order kinetics were calculated from plotting of ln (q_e_ − q_t_) vs. t and (t/q_t_) vs. t, respectively: see Figure S3 (Supplementary Material). Table S4 (Supplementary Material) summarizes the obtained information. The R^2^ values confirmed that the Lagergren pseudo-second-order kinetic was the acceptable kinetic model, and this explains the chemical adsorption type which occurs through sharing between the used adsorbent materials and the dissolved ions in addition to the physical one [53]. The experimental adsorption capacity q_e_ (mg/g) was 1.06 mg/g for the removal of ammonia and 0.295 mg/g for the removal of iron, while the calculated values were 1.469 mg/g and is 0.36 mg/g for ammonia and iron removal, respectively.

### 2.6. Binding (Complexation) Energy of Grafted Hydrogel with Cations

In fact, we have tested three positions, specifically the places with the richest charge density. Our result for this step is presented in Figure S4 (Supplementary Material). Our calculations confirmed that Structure 3 is the most stable structure. Structure 3 is more stable than Structure 1 by 18.71 kcal/mol and Structure 2 by 10.19 kcal/mol. Structures 1 and 2 showed one hydrogen bond between the oxygen atom of the grafted hydrogel and one hydrogen of the ammonium ion, while Structure 3 showed three hydrogen bonds between the grafted hydrogel and the ammonium ion. This strong hydrogen bonding awards an extra stability of structure 3 over structures 1 and 2. Iron (II) and Iron (III) are optimized by locating them in the same position of ammonium cation as in structure 3.

**Figure 7:**
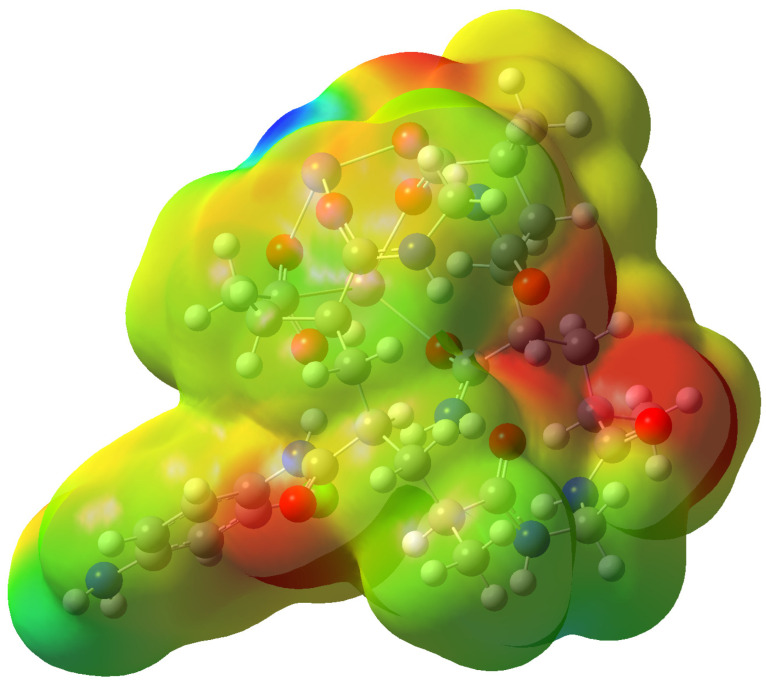
Molecular electrostatic potential (MESP (−0.055 to 0.055)) of grafted hydrogel at the B3LYP/6-311G(d,p) level of theory, Red represents negative, blue represents positive and green is in between.

The molecular electrostatic potential is exhibited in Figure 7. The red color represents a negative area, the blue expresses a positive area, and the green color is an area between them. The MSEP demonstrated the positivity of the nitrogen of the amide group that attacks the para position of the substituted aniline. Also, the MESP confirmed the best position for the metal cation complexation. This position is showed in the red color and characterized by the three oxygen atoms that are arranged in the manner of a hole that allows the metal cation to reside on it.

The corrected and uncorrected binding energies of the hydrogel–metal cation complexes in the gas phase were also calculated, at the same level of theory of the calculation used in that article, using the basis set superposition error (BSSE), which uses the counterpoise correction approach [54] and is summarized in Table 3. The binding energy E_bind_ for each hydrogel–metal cation complex is obtained according to the following equation:

ΔE_bind_ = E_complex_ − (E_ion_ + E_hydrogel_) (10)

**Figure 8:**
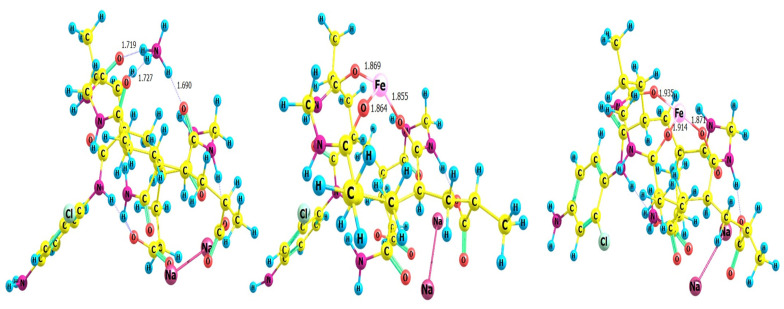
The optimized structures of hydrogel–metal complexes (left NH^4+^, middle Fe^2+^, right Fe^3+^) at the B3LYP/6-311G(d,p) level of theory.

The optimized structures for the hydrogel–metal cation NH_4_^+^, Fe^2+^ and Fe^3+^ complexes are validated in Figure 8. The hydrogens of the ammonium cation showed a strong hydrogen bonding between them and the three oxygens of the hydrogel. The complexes with Iron (II) and (III) expressed very strong bonds between the metal cations and the oxygens of the grafted copolymer. It is confirmed from the values of the bond lengths between the iron and the hydrogel that the metal ion occupies the center of the triangle arranged by the three oxygens. This is clear from the nearly identical values of the distances between the cation and the oxygen atoms. In the case of Iron (III), the bond lengths are larger than in the case of Fe^3+^, since it has a smaller atomic radius than Fe^2+^. The uncorrected and corrected complexing energy between the two monomers (hydrogel and metal cations) are presented in Table 3. It is clear that the complexation energy was strongly affected from the results of the BSSE correction. Thus, these corrections should be considered. The data in Table 3 show the stronger interaction between the hydrogel with iron cations than an ammonium cation. This due to the fitting of the size of the iron to the cavity of the three oxygens in the grafted copolymer. The complexation energy of Iron (III) is more than double that of Iron (II) due the strong interaction between Fe^3+^ because of the higher charge.

### 2.7. Regeneration Study

**Figure 9:**
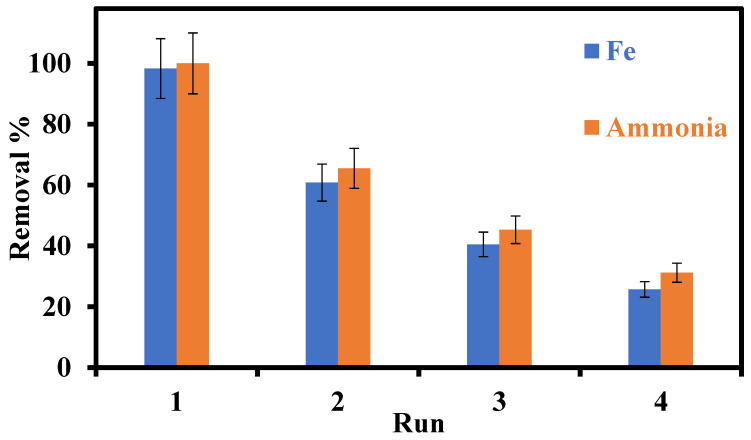
Adsorption of iron and ammonia by graft under 4 runs of recycling.

Reusing adsorbent materials has drawn a lot of interest as a tactic to limit waste production and cut costs associated with processing. Four successive recycling tests were carried out employing the graft to evaluate their potential for numerous applications, making use of the ideal conditions created in this work. To reduce pollution, the adsorbents underwent a washing process using only distilled water after each adsorption cycle. They were then dried at room temperature and used for the next adsorption cycle. The data are summarized in Figure 9. The removal of iron and ammonia decreased in Runs 2, 3 and 4.

### 2.8. Swelling Studies

A total of 1 g of the graft was chosen for the swelling studies. Over time, the weight of the graft increased because of the swelling process (Table 4).

## 3. Conclusions

Groundwater is a one of the major sources for drinking water throughout the world. It is known that water is necessary for life, and that some countries, even where there are rivers, need to use groundwater. The researchers’ mission is to check this water to make it suitable for use. Research has shown the presence of ammonia and iron in groundwater. Therefore, researchers must find the fastest and least expensive way to remove ammonia and iron from water. The polymeric compounds used in this research fitted the two criteria of being easy to prepare and quite inexpensive. And the obtained results showed their efficiencies for removing ammonia (100%) and iron (98.3%) from water. DFT methods have been used to obtain the stationary states of the hydrogel and grafted hydrogel and calculate the binding energies of the grafted hydrogel with the studied cations. The calculated complexation energy was corrected using the BSSE method. Our grafted hydrogel revealed a strong selectivity towards the studied cations. The calculated complexation energy by the DFT method decreased in the following order: Fe^3+^, Fe^2+^ and NH_4_^+^. It has been confirmed by the calculated binding energy that the grafted hydrogel is an excellent extractor for different cations from polluted water. Thus, our hydrogel shows an environmental application as a water treatment.

## 4. Materials and Methods

### 4.1. Materials

The 3-chloroaniline 99%, acrylic acid 99%, N, N/-methylenebisacrylamide (cross-linker) 99.5%, potassium persulphate (PPS) 99% and dimethyl formamide (DMF) 99.9% were products of Sigma Aldrich. Acrylamide 98% was procured from the Oxford Company. Hydrochloric acid 33% and sodium hydroxide pellets 98% were products of the Prolabo Chemical Company. Acetone 99.5% and methanol 99.5% were provided by El-Nasr Pharmaceutical, Chemical Company.

### 4.2. Preparation of Polyacrylate–Polyacrylamide Hydrogel Grafted with 3-Chloroaniline

The polyacrylate–polyacrylamide hydrogel was prepared by the method used in our previous article [27]. The solution of 3-chloroaniline was prepared by dissolving 1 mL of miscible 3-chloroaniline in 25 mL acidified water with 0.5 mol/L HCl. After that, 1 g of the polyacrylate–polyacrylamide hydrogel was added separately to the 3-chloroaniline solution, and then the mixture was left overnight to complete swelling. A K_2_S_2_O_8_ solution (1 g: 25 mL water) was added to the mixture in an ice bath (0–10 °C). The grafting was started, and the product was left overnight. The grafted hydrogel was separated, washed with methanol several times, washed with distilled water and dried under vacuum at 70 °C.

### 4.3. Instrumental Techniques

#### 4.3.1. FTIR

The functional groups in grafted hydrogels and hydrogel are identified using infrared by FT-IR spectroscopy (Vertex 70 Bruker) in the range of 400–4000 cm^−1^ with the mode of reflection at a 4 cm^−1^ resolution at room temperature.

#### 4.3.2. Morphological Studies

The Pananlytical Empryan X-ray diffractometer 202,964 was used to examine the XRD patterns of the grafted hydrogel. The scanning area was 5–80°. The scanning electron microscopic (SEM) images were captured using JEOL JSM-6510LA (SEM), beam energy: 20–30 kV, working distance: 11.1–12.2 mm.

#### 4.3.3. Transmission Electron Microscope (TEM)

The measurements were carried out using a carbon-coated copper grid as a photographic plate of the transmission electron microscope.

#### 4.3.4. Thermogravimetric Analysis (TGA)

TGA analysis was obtained by using the Shimadzu TGA-50H detector with a platinum cell, a nitrogen atmosphere and a 20 °C/min flow rate in the range of 38 °C to 725 °C.

### 4.4. Sampling of Groundwater

The groundwater in this investigation was collected from Al Garnos and Shoulqam (Al-Minya, Egypt). The samples were collected in polypropylene containers after being washed with diluted HNO_3_ and rinsed with distilled water. On various dates, we collected 10 samples from Shoulqam (from 2 wells) and 15 samples from Al Garnos (from 3 wells). The parameters of the groundwater samples are shown in Table 5 before and after treatments, and they demonstrate a slight reduction in turbidity, chloride content and overall hardness and a significant decrease in TDS.

#### 4.4.1. Measurement of Ammonia Concentration

The indophenol technique was used in the YSI ammonia test. In the presence of chlorine, ammonia interacts with alkaline salicylate to generate a green-blue indophenol complex. Catalysts were used to ensure full and rapid color growth. The reagents were delivered in the form of two tablets for maximum convenience. The test was carried out by placing one of each pill in a sample of water. A YSI photometer was used to calculate the color intensity produced which is proportional to the concentration of ammonia.

#### 4.4.2. Measurement of Iron Concentration

The YSI iron LR approach employed a single tablet reagent comprising 3-(2-pyridyl)-5,6-bis (4-phenyl-sulphonic acid)-1,2,4-triazine (PPST) combined with a decomplexing/reducing agent in an acid buffer. The study was carried out by placing a tablet in the tested sample. The decomplexing/reducing agent breaks down the iron weak complex and converts it to ferrous ion. In other words, the iron weak complexes were reduced to iron (II) cations by the aid of the reducing agent. These Ferrous ions were identified by formation of a pink color with PPST. A YSI photometer was used to determine the color intensity which is proportional to the concentration of iron.

#### 4.4.3. Computational Details

The ground states of the studied compounds have been investigated using Gaussian 03 [55]. Full optimization was performed using density functional theory using the B3LYP/6-311G(d,p) level of the theory [56,57,58,59]. Moreover, the graft iron complexes were calculated using the LANL2DZ [60,61] basis set for the iron atom and 6-311G(d,p) basis set for the remaining atoms using the same function. The frequency calculations were computed at the same level of theory for ensuring the minima structure and no imaginary frequencies have been observed. The molecular electrostatic (MEP) potential and electron density for the ligand in 3D plots were calculated using the same level of theory. The computational calculations were performed in the following order. 1- Optimization followed by frequency calculations for the copolymer. 2- Investigation of the natural bond orbitals (NBO) for the copolymer to locate the grafting position. 3- Optimization followed by frequency calculations for the hydrogel (grafted copolymer). 4- Calculating the molecular electrostatic potential to find the best place for the cations on the grafted copolymer. 5- Finally, the complexing (binding) energy of different cations with the grafted copolymer has been obtained.

## Figures and Tables

**Table 1:** Standard deviation.

Pollutants	Samples Removal					Average	SD
	S1	S2	S3	S4	S5		
**Iron**	98.3	100	96	97	95.7	97.4	1.77341479
**Ammonia**	100	99.4	98.5	96.8	97.2	98.38	1.37549991

**Table 2:** Parameters for the adsorption of ammonia and iron using different isotherms.

Model	Parameter	Parameter Value	
		Ammonia	Iron
**Langmuir**	Q_m_ (mg g^−1^)	3.52	0.55
	B	15.439	35.1
	R^2^	0.9494	0.9877
**Freundlich**	N	3.4	4.25
	K_f_	2.06	24
	R^2^	0.8997	0.9592
**Temkin**	B_T_ (J/mol)	0.5739	0.0904
	K_T_ (L/g)	2.76	85.39
	R^2^	0.8502	0.9354

**Table 3:** The binding energies (ΔE_bind_ and ΔE_bind_ with (BSSE) correction, in kcal/mol).

Ion	Complexation Energy kcal/mol	
Method	(Raw)	(Corrected) BSSE
**Iron (II)**	−410.49	−401.65
**Iron (III)**	−990.86	−920.7
**Ammonium**	−74.6	−71.1

**Table 4:** Swelling percentage of the graft with water at different time periods.

Time (h)	1 h	6 h	12 h	24 h	48 h	72 h
**Swelled weight of graft**	1.05 g	1.33 g	1.55 g	1.98 g	2.31 g	2.62 g
**Swelling %**	5%	33%	55%	98%	131%	162%

**Table 5:** Groundwater properties.

Parameter	Before Treatment	After Treatment					Average	SD	Standard Values
		S1	S2	S3	S4	S5			
Turbidity	1.3	1	0.9	1.1	0.8	0.8	0.92	0.13038405	1.0 NTU
Chlorides	140	130	133	128	131	129	130.2	1.92353841	250 mg/L
Alkalinity	320	320	318	319	317	316	318	1.58113883	500 mg/L
TDS	710	650	654	653	660	658	655	4	1000 mg/L
Total hardness	280	250	240	260	244	264	251.6	10.2371871	500 mg/L
Ca hardness	130	120	124	116	128	118	121.2	4.81663783	350 mg/L
Mg hardness	150	130	124	132	136	122	128.8	5.76194412	150 mg/L
Ammonia	0.8	0	0.001	0.002	0.003	0.0015	0.0015	0.00111803	0.5 mg/L
Iron	1.2	0.02	0.01	0.015	0.013	0.014	0.0144	0.00364692	0.3 mg/L

## Data Availability

All of the datasets that support the findings and underlie the conclusion in this paper should be available to all readers upon request from the authors.

## Supplementary Materials

The following supporting information can be downloaded at: https://www.mdpi.com/article/10.3390/gels9100781/s1, Figure S1: Effect of temperature on the removal of iron (a) and ammonia (b) efficiency at different doses of grafted hydrogel. Van’t Hoff plot for the adsorption of iron (c) and ammonia (d) on grafted hydrogel. Figure S2: Temkin isotherm for iron (a) and ammonia (b) removal by grafted hydrogel. Figure S3: Pseudo-first-order kinetic model of iron (a) and ammonia (b) removal. Pseudo-second-order kinetic model of iron (c) and ammonia (d) removal. Figure S4: The optimized structures of complexes of the grafted hydrogel with the NH_4_^+^ ion in different positions at the B3LYP/6-311G(d,p) level of theory. Table S1: IR bands and their assignments for hydrogel and grafted hydrogel. Table S2: TGA data of the fabricated samples (hydrogel and grafted hydrogel). Table S3: Thermodynamic parameters. Table S4: Kinetic models’ parameters data.
