# Supplementary material for: Novel Grafted Hydrogel for Iron and Ammonia Removal from Groundwater: A Synthesis and Computational Chemistry Study
Source: Gels. 2023 Sep 25;9(10):781. doi: 10.3390/gels9100781 (PMC10606057; doi:10.3390/gels9100781)
Supplement: Supplementary file 1 [file gels-09-00781-s001.zip › gels-2566548-supplementary.pdf]

Supplementary Material

Novel Grafted Hydrogel for Iron and Ammonia Removal from Groundwater: A Synthesis and Computational ChemistryStudy

Hanafy M. Abd El-Salam 1, Ali M. El Shafey 1, Abdelouahid Samadi 2 and Mahmoud K. Abdel-Latif 2,3,*

1 Polymer Research Laboratory, Department of Chemistry, Faculty of Science, Beni-Suef University, Beni-Suef City 62514, Egypt; hanafya@yahoo.com (H.M.A.E.-S.); mly390861@gmail.com (A.M.E.S.)

2 Chemistry Department, Collage of Science, United Arab Emirates University, Al-Ain 15551, United Arab Emirates; samadi@uaeu.ac.ae

3 Chemistry Department, Faculty of Science, Beni-Suef University, Beni-Suef City 62514, Egypt

* Correspondence: mahmoudkorani@uaeu.ac.ae

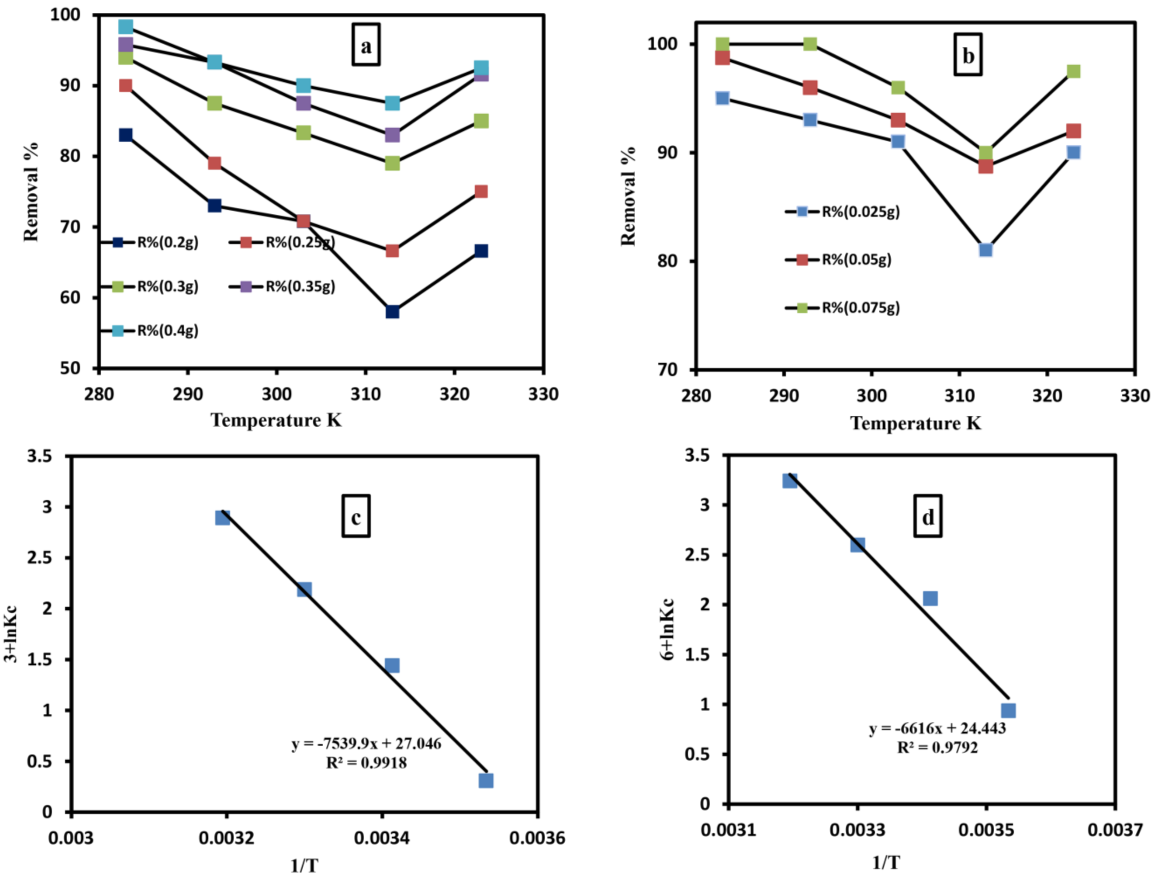

Figure S1: Effect Temperature on the removal iron(A) and ammonia(b) efficiency at different dose of grafted hydrogel. Van't Hoff plot for the adsorption of iron (c) and ammonia (d) on grafted hydrogel

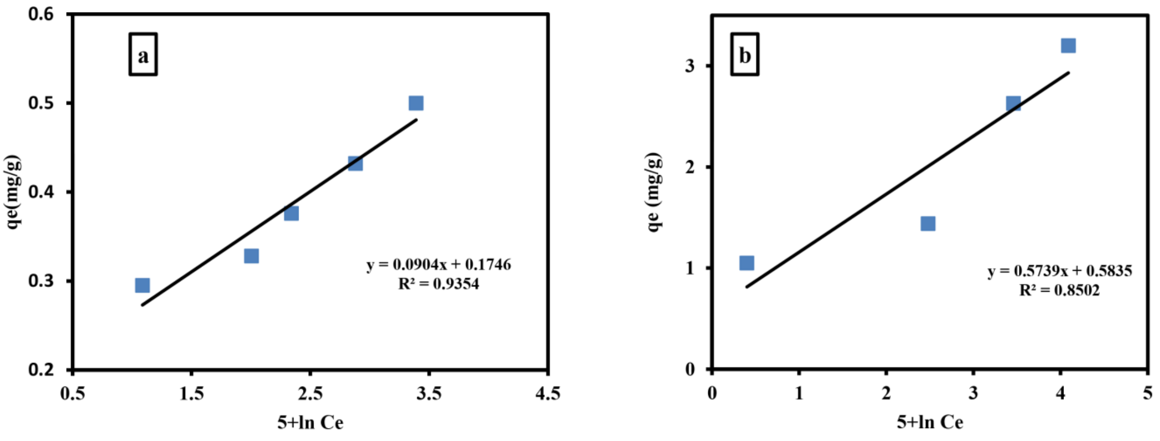

Figure S2: Temkin isotherm for iron (a) and ammonia (b) removal by grafted hydrogel

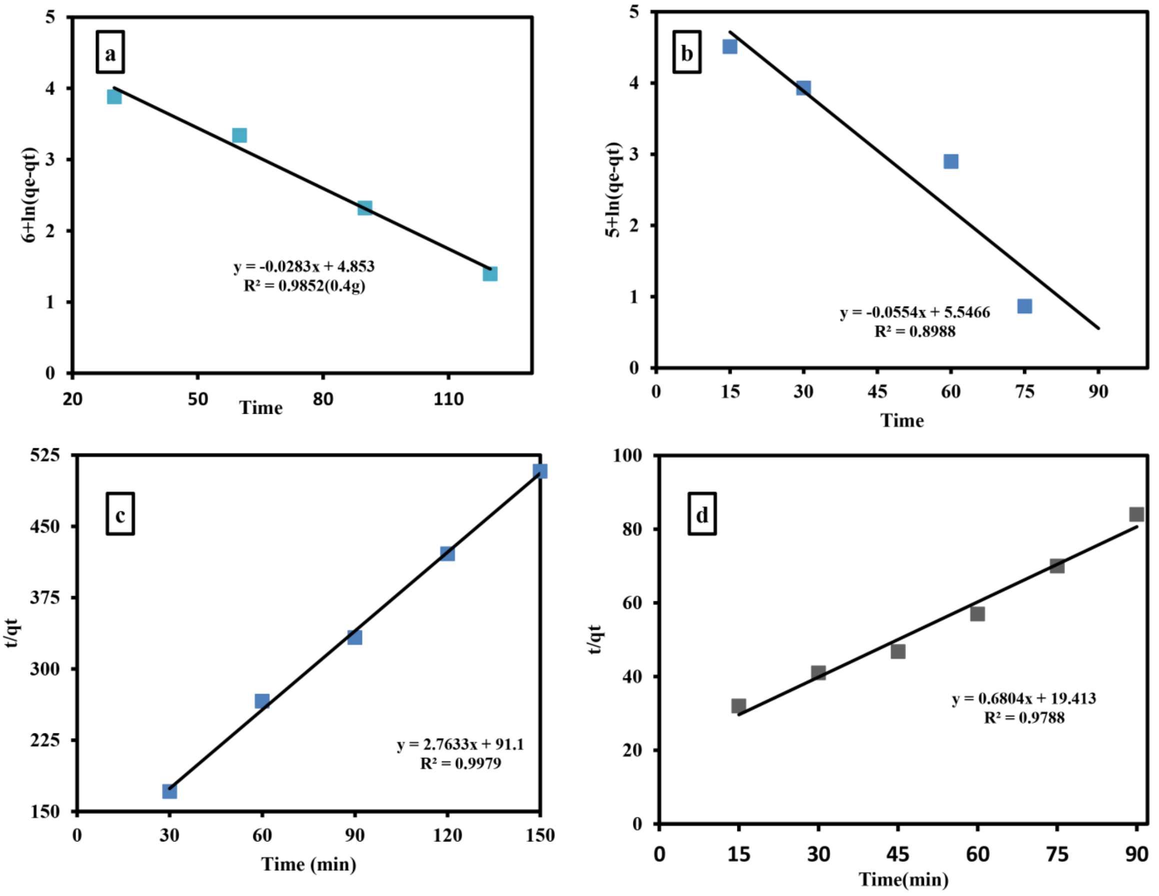

Figure S3: Pseudo-first-order kinetic model of iron (a) and ammonia (b) removal. Pseudo-second-order kinetic model of iron (c) and ammonia (d) removal.

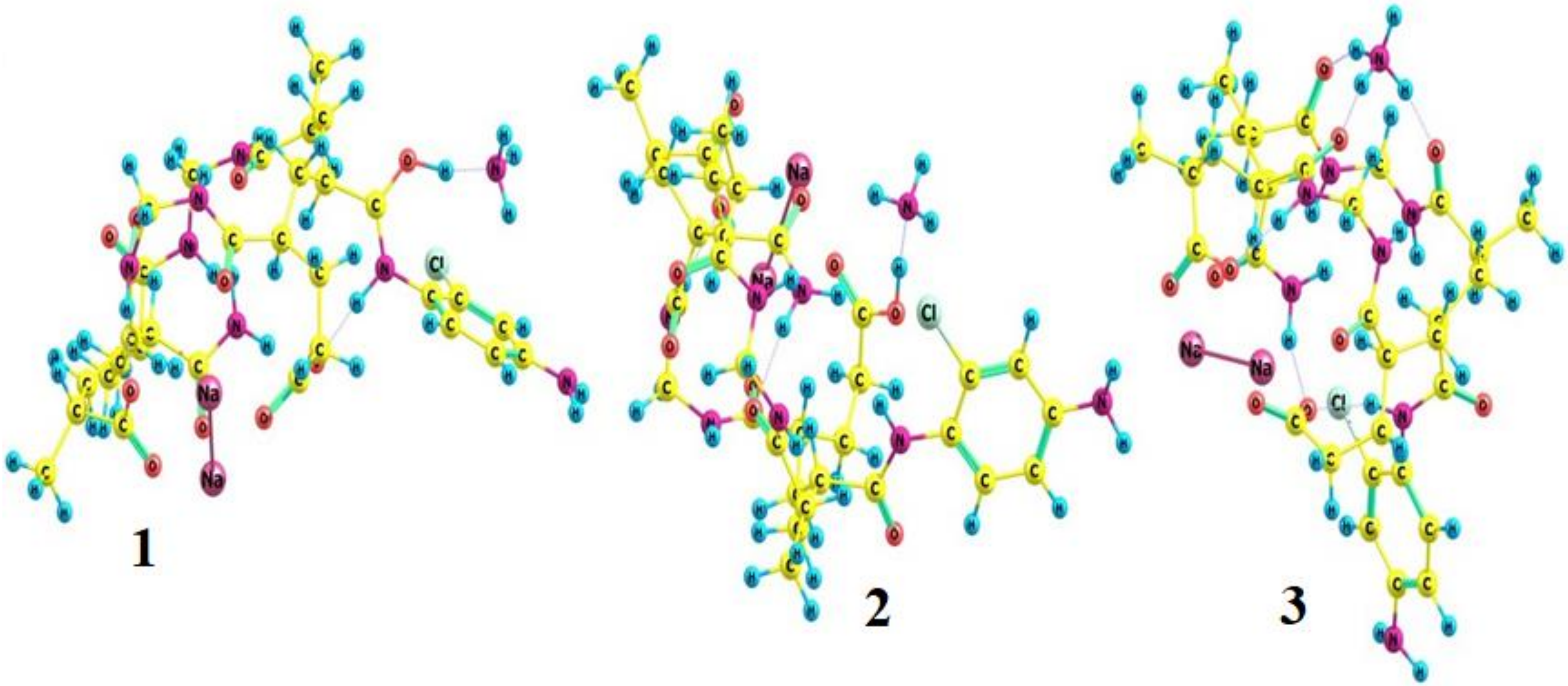

Figure S4: The optimized structures of complexes of the grafted hydrogel with the NH_4^+ ion in different positions at the B3LYP/6-311G(d,p) level of theory.

Table S1: IR bands and their assignments for hydrogel and grafted hydrogel.

Wave number cm^{-1}<br>Hydrogel	Wave number cm^{-1}<br>Grafted hydrogel	Assignments	Ref.
612^{vw}<br>777^{vw}	608^{m}<br>783^{s}	Stretching vibration of substituted benzene ring	a*
1115^{w}<br>1176^{w}	1110^{w}<br>1180^{w}	C-O and C-N stretching	b*<br>c*
1334^{w}<br>1449^{m}	1330^{w}<br>1447^{m}	CH_2 bending	d*
-<br>1656^{s}	1590^{w}<br>1657^{s}	C=O, C=C and/or adsorbed H_2O molecules and –COONa	e*
-<br>2376^{w}	2069^{m}<br>2382^{w}	Overtone and combination bands	
2516w<br>2859^{w}<br>2926^{m}	-<br>-<br>2931^{s}	Aliphatic CH	f*
3422^{b}	3410^{b}	Stretching vibration of OH and/or NH_2 free or bonded	g*
w=weak, b=broad, s=strong, m=medium			

a*: (Abd El-Mageed, Abd El-Salam, Abdel- Latif, & Mustafa, 2018)
b*: (Monteiro & Neves, 2014)

c*: (Paiva, 2007)

d*: (Silverstein, Bassler, & Morill, 1974)

e*: (Reddy & Lee, 2007)

f*: (Asabe & Bashar, 2016)

g*: (Rojas & Carlos 2019)

Table S2: TGA data of the fabricated samples (hydrogel and grafted hydrogel).

TGA parameters for polymeric samples				Comments
Hydrogel		grafted hydrogel		
Midpoint ^{0}C	Approx. Wt. loss %	Midpoint ^{0}C	Approx. Wt. loss %	
73	4	95	2	Moisture losing
140	12	116	8	Bonded water loss
233	21	220	13	
335	38	367	17	Series of fragmentation of the polymer backbone depend on the bond strength between atoms
371	50	-	-	
403	52	-	-	
422	63	441	55	
466	72	460	60	
703	90	725	60	Complete degradation with carbonic residue is about 10% for hydrogel but for graft the residue is about 40%.
-		821	60	
-		918	60	
-		952	60	

Table S3: Thermodynamic parameters

Thermodynamic parameters		G (kJ mol^{-1})Δ	
		Removal of ammonia	Removal of iron
Temperature K	283	-1076.26	-6332.12
	293	-1051.82	-3795.64
	303	-1021.42	-2042.85
	313	-912.508	-280.085
ΔH (kJ mol^{-1})		-55.005	-62.687
S (J mol^{-1} K $^{-1}$)Δ		-203.219	-244.9

Table S4: Kinetic models parameters data.

**Model**	**Parameter**	**Parameter value**	
		**Ammonia removal**	**Iron removal**
**Pseudo-first order**	k_1(min^{-1})	0.0554	0.0283
	q_e (mg g^{-1})	254.6	128
	R^2	0.8988	0.98
**Pseudo-second order**	k_2(min^{-1})	0.023	7.71
	q_e(exp) (mg g^{-1})	1.06	0.295
	qe(cal) (mg/g)	1.469	0.36
	R^2	0.9788	0.9979
